# Supplementary material for: Content-rich biological network constructed by mining PubMed abstracts
Source: BMC Bioinformatics. 2004 Oct 8;5:147. doi: 10.1186/1471-2105-5-147 (PMC528731; doi:10.1186/1471-2105-5-147)
Supplement: Additional File 2 — The original results of the above study (non-essential files are deleted to keep the file size under the limit set by BMC bioinformatics). [file 1471-2105-5-147-S2.bz2 › chilibotAdditionalFile2/dip50/513ID10344732E10815/html/CCND1_AR.html]

 


 **CCND1** and **AR** 
  
Found 122 abstracts in PubMed, retrieved 50.  
 

 What does Google say? 
 PDF only 
| .edu only 

---

**Interactive relationship** (e.g. stimulation, inhibition, etc)

**Stimulatory relationship**- **AR**  transgenic mice display increased Ras, Erk1 2, cyclin D  [ **CCND1** ]  CDK4, and cyclin E CDK2 activity and G1 S progression in pancreatic duct cells.  Ref: 12055597 Gastroenterology, 2002
- Finally, microarray analyses were performed that showed increased expression of Bcl  [ **CCND1** ]  3, an NFkappaB IkappaB factor, was correlated with AIS  [ **AR** ] .  Ref: 12538159 Ann N Y Acad Sci, 2002
- In contrast,  **AR** , a specific ErbB1 ligand, induced sustained ERK1 2 and Elk1 phosphorylation, increased cyclin D1  [ **CCND1** ]  mRNA and protein, and increased DNA synthesis activity.  Ref: 12869389 Circ ResCirc Res, 2002
- Down regulation of bcl  [ **CCND1** ]  2 protein and mRNA by DHT was inhibited by coincubation with the antiandrogen bicalutamide, an agent that competitively inhibits binding of DHT to androgen receptor  [ **AR** ] .  Ref: 12629413 J Urol, 2003
- Treatment of LY  **ar**  cells with the MEK inhibitors PD 98059, U0126, and PD 184352 led to a loss of phosphorylated ERK1 and ERK2, a reversal of nuclear p50 homodimer DNA binding, and a decrease in Bcl  [ **CCND1** ]  2 protein expression.  Ref: 12801933 J Biol Chem< MedlineTA>J Biol Chem, 2003
**Neutral relationship**- ... a sustained ERK Elk1 activation could be the major signaling cascade for  **AR**  induced upregulation of cyclin D1  [ **CCND1** ]  protein in HTASMCs.  Ref: 12869389 Circ ResCirc Res, 2003
- In NPC, expression of p16 was positively correlated, while bcl  [ **CCND1** ]  2 negatively correlated with the  **AR**  P <.  Ref: 11776648 Zhonghua Zhong Liu Za Zhi, 2000
- We demonstrate that cyclin D1  [ **CCND1** ]  directly binds the N terminus of the androgen receptor  [ **AR** ] .  Ref: 11714699 J Biol Chem, 2002
- Hormone resistance is typical for tumors from urothelial, basal and neuroendocrine PC cells, glandular epithelium cells which lost androgen receptors  **AR**  and tumors consisting of cells which retain  **AR**  but simultaneously express Bcl  [ **CCND1** ]  2 and or p53 genes.  Ref: 11569231 Urologiia< MedlineTA>Urologiia, 2002
- Differential expression of BCL  [ **CCND1** ]  2 family proteins may be responsible for the higher  **AR**  observed in ALK ALCL and provides a possible biological explanation for the better prognosis reported for patients with ALK ALCL.  Ref: 11485911 Am J Pathol, 2001
- In addition to the effects on cell proliferation, FGF2 maintains the survival of  **AR**  PC3 clones through a positive modulation of the Bcl  [ **CCND1** ]  2 protein and down modulates  **AR**  protein expression, allowing the escape of selected clones from androgen regulation.  Ref: 12430142 Prostate, 2002
- We therefore assessed expression levels of the anti apoptotic proteins BCL  [ **CCND1** ]  2 and BCL  [ **CCND1** ]  XL and the pro apoptotic proteins BAX and BCL  [ **CCND1** ]  XS in T null cell ALCL using immunohistochemical methods and correlated the findings with ALK expression and apoptotic rate  **AR** , the latter assessed by a modified Tdt mediated dUTP nick end labeling assay.  Ref: 11485911 Am J Pathol, 2001
- CONCLUSION In the presence of an active  **AR** , the combined production of FGF2 and FGF BP may play an important role in the progression of prostate cancer through the selection of  **AR**  clones expressing high levels of Bcl  [ **CCND1** ]  2.  Ref: 12430142 Prostate, 2002
- Pretreatment with PD98059 suppressed  **AR**  induced cyclin D1  [ **CCND1** ]  mRNA and protein.  Ref: 12869389 Circ ResCirc Res, 2002
- By contrast, cyclin D1  [ **CCND1** ]  completely abolished androgen receptor  [ **AR** ]  activity, even in the presence of potent androgen receptor  [ **AR** ]  co activators.  Ref: 11714699 J Biol Chem, 2002

**Non-interactive relationship** (e.g. studied together, co-existance, homology, etc.)

- We investigated the role of NF kappaB1 p50 homodimers in the expression of Bcl  [ **CCND1** ]  2 in two murine B cell lymphoma cell lines LY as, an apoptosis proficient line with low Bcl  [ **CCND1** ]  2 protein expression and no nuclear NF kappaB activity, and LY  **ar** , a nonapoptotic line with constitutive p50 homodimer activity and 30 times more Bcl  [ **CCND1** ]  2 protein expression than LY as.  Ref: 11567031 J Biol Chem, 2001
- Cyclin D1  [ **CCND1** ]  mechanism and consequence of androgen receptor  [ **AR** ]  co repressor activity.  Ref: 11714699 J Biol Chem, 2002
- After the onset of progesterone production, Bcl  [ **CCND1** ]  2 levels decrease and Ki67 levels and androgen receptor  [ **AR** ]  expression in stromal cells disappear resulting in cell disintegration and menstruation.  Ref: 12186988 Gynecol Obstet Invest, 2002
- They become Bcl  [ **CCND1** ]  2 and or p53 positive while part of them lose  **AR** .  Ref: 11569231 Urologiia< MedlineTA>Urologiia, 2002
- We have shown previously that cyclin D1  [ **CCND1** ]  functions as a co repressor to inhibit ligand dependent androgen receptor  [ **AR** ]  activation.  Ref: 11714699 J Biol Chem, 2002
- According to 3 year follow up, the survival rate was higher in NPC patients with  **AR**  > 2.0 and negative bcl  [ **CCND1** ]  2 expression.  Ref: 11776648 Zhonghua Zhong Liu Za Zhi, 2000
- In contrast, expression of differentiation specific proteins prostate specific antigen and androgen receptor  [ **AR** ]  or proliferative regulatory proteins c myc, cyclin D1  [ **CCND1** ] , p27 were down regulated by hypoxia.  Ref: 12481256 Prostate, 2003
- Using terminal deoxynucleotidyl transferase mediated dUTP biotin nick end labeling Tunel and immunohisytochemistry S P method, the apoptotic rate  **AR**  and expression of p16 and bcl  [ **CCND1** ]  2 protein were examined.  Ref: 11776648 Zhonghua Zhong Liu Za Zhi, 2000
- Flow cytometric stains for antiapoptotic Bcl  [ **CCND1** ]  2 and Bcl  [ **CCND1** ]  xL showed that neither factor was well correlated with AIS  [ **AR** ] , although both were increased transiently upon T cell activation.  Ref: 12538159 Ann N Y Acad Sci, 2002
- To investigate the mechanism of the AI growth displayed by the GOF p53 mutants, Western blotting or ELISA were used to examine the expression of the androgen receptor  [ **AR** ]   **AR** , the  **AR**  regulated prostate specific antigen PSA, as well as Akt and Bcl  [ **CCND1** ]  2 under androgen depleted conditions.  Ref: 12727844 Cancer Res, 2003
- In a previously published report, we described the NF kappaB status for two murine B cell lymphoma cell lines, LY as apoptosis sensitive and LY  **ar**  apoptosis refractory, and provided evidence that NF kappaB1 p50 homodimers contribute to the expression of Bcl  [ **CCND1** ]  2 in the LY  **ar**  line.  Ref: 12801933 J Biol Chem< MedlineTA>J Biol Chem, 2003
- We found that the allele frequencies of both polymorphisms did not differ between the SID and control groups 0.65 0.35 versus 0.72 0.28 for UCP 1 Bcl  [ **CCND1** ]  I, and 0.89 0.11 versus 0.93 0.07 for beta3  **AR**  Trp64Arg in SID victims versus controls, respectively.  Ref: 12029453 Eur J Pediatr, 2002
- The expression of prostate marker genes, including 5alpha reductase, prostate specific antigen, prostatic acid phosphatase and androgen receptor  [ **AR** ] , and the anti apoptotic bcl  [ **CCND1** ]  2 gene were markedly reduced, but the transcription of apoptosis related caspase 3 gene showed a dose dependent increase in lemnabourside treated LNCaP cells.  Ref: 11837251 Life Sci, 2002
- To determine whether the UCP 1 Bcl  [ **CCND1** ]  I polymorphism and or the Trp64Arg variant of beta3  **AR**  are associated with the occurrence of SID, we determined the allele frequencies of these polymorphisms in 53 Austrian SID victims and 54 controls by nested PCR and restriction digestion using DNA extracted from Guthrie cards.  Ref: 12029453 Eur J Pediatr, 2002
- and 3 the expression of p53, pRB, Bcl  [ **CCND1** ]  2, Bax, p16, p21, p27, cyclins A, B, D1, E, p34 cdc2, and the androgen receptor  [ **AR** ]   **AR** , by western blot analysis.  Ref: 12147712 Mol Pathol, 2002
- The study of the prognostic criteria of hormone resistant prostatic cancer PC by specifying expression of androgen receptor  [ **AR** ]  protein as well as Bcl  [ **CCND1** ]  2 and p53 proteins, apoptosis regulators, has demonstrated that tumor cells of hormone sensitive and hormone resistant PC forms have different variants of immunophenotype.  Ref: 11569231 Urologiia< MedlineTA>Urologiia, 2002
- Bcl  [ **CCND1** ]  2 and androgen receptor  [ **AR** ]  gene expression in androgen independent subclone derived from mouse androgen dependent cells.  Ref: 12197229 Cancer Invest, 2002
- We examined the impact of beta  **AR**  blockade with metoprolol on myocardial remodeling, apoptosis, pro apoptotic Fas, Fas ligand, Bax, and Bcl  [ **CCND1** ]  X S and anti apoptotic Bcl  [ **CCND1** ]  X L and Bcl  [ **CCND1** ]  2 gene expression, and Bcl  [ **CCND1** ]  X L and Bcl  [ **CCND1** ]  X S protein in post infarction HF in rats.  Ref: 12738230 J Mol Cell Cardiol, 2003
- We conclude that the p50 homodimer is capable of transcriptional activation of the bcl  [ **CCND1** ]  2 gene and suggest that its nuclear activity contributes to the expression of bcl  [ **CCND1** ]  2 in LY  **ar**  cells.  Ref: 11567031 J Biol Chem, 2001
- Analysis of protein expression revealed only a weak association between Bcl  [ **CCND1** ]  2 and  **AR** .  Ref: 12098003 Neoplasma, 2002
- We conclude that adrenergic activation, such as occurs in HF, increases pro apoptotic Bcl  [ **CCND1** ]  X S expression via the beta 1  **AR** . beta  **AR**  blockade in HF reduces myocardial apoptosis.  Ref: 12738230 J Mol Cell Cardiol, 2003
- The status of antisense targeting of genes relevant to prostate cancer, including bcl  [ **CCND1** ]  2, bcl  [ **CCND1** ]  xL, clusterin, androgen receptor  [ **AR** ]   **AR**  and IGFBPs, are reviewed.  Ref: 12400997 Cancer Metastasis Rev, 2002
- The mechanisms of action may involve downregulation of the androgen receptor  [ **AR** ] , induction of apoptosis by way of inhibition of the bcl  [ **CCND1** ]  2 gene, and increased expression of p53.  Ref: 12350462 Urology, 2002
- No association was found between p21 expression and p53, bcl  [ **CCND1** ]  2, and the androgen receptor  [ **AR** ]  protein expression in bone metastases of patients with AIPC.  Ref: 11895908 Clin Cancer Res, 2002
- The status of antisense targeting of several genes, including bcl  [ **CCND1** ]  2, bcl  [ **CCND1** ]  xL, clusterin, androgen receptor  [ **AR** ]  and IGFBPs, relevant to prostate and other cancers, are reviewed.  Ref: 12643471 Curr Drug Targets, 2003
- We observed increased expressions of genes including NADH dehydrogenase, the GLUT4 glucose transporter, Na K ATPase, sarcolemmal K ATP channels, and Bcl  [ **CCND1** ]  xl in A 1  **AR**  overexpressing hearts.  Ref: 12388787 Physiol Genomics, 2002
- We investigated bcl  [ **CCND1** ]  2 and androgen receptor  [ **AR** ]   **AR**  expression in an androgen independent subline derived from mouse androgen dependent mammary carcinoma cells.  Ref: 12197229 Cancer Invest, 2002
- These data indicate that the increased expression of Bcl  [ **CCND1** ]  2 protein plays an important role in acquiring tolerance for the testosterone ablation, although it is not related to  **AR**  gene alteration.  Ref: 12197229 Cancer Invest, 2002
